# Osimertinib as Salvage Therapy in Advanced Non‐Small Cell Lung Cancer After Aumolertinib Resistance With T790M Mutation: A Case Report

**DOI:** 10.1002/ccr3.70219

**Published:** 2025-02-17

**Authors:** Yongtong Tang, Jilan Xu, Qingle Chen, Jianping Lu, Li Ren, Taihe Lan, Taihua Liao, Xiaoling Chen, Hui Yi, Jun Zhang, Jie Huang

**Affiliations:** ^1^ Department of Pulmonary and Critical Care Medicine People's Hospital of Shangyou County Ganzhou China; ^2^ Department of Medical Imaging People's Hospital of Shangyou County Ganzhou China; ^3^ Department of Pathology People's Hospital of Shangyou County Ganzhou China; ^4^ Guangdong Lung Cancer Institute, Guangdong Provincial Key Laboratory of Translational Medicine in Lung Cancer, Guangdong Provincial People's Hospital (Guangdong Academy of Medical Sciences) Southern Medical University Guangzhou China; ^5^ Department of Oncology Guangdong Provincial People's Hospital Ganzhou Hospital, Ganzhou Municipal Hospital Ganzhou China

**Keywords:** aumolertinib, epithelial growth factor receptor, osimertinib, resistance

## Abstract

The third generation of epidermal growth factor receptor (EGFR) tyrosine kinase inhibitors (TKIs) is recommended universally as the standard treatment for non‐small cell lung cancer (NSCLC) carrying the *EGFR* T790M mutation. With the approval of multiple third‐generation EGFR‐TKIs, questions have arisen regarding the differences in their efficacy and how to select the most appropriate agent for individual patients. The study reports a case of an advanced NSCLC patient with multiple brain metastases. The patient initially received the pemetrexed plus carboplatin (PC) regimen for 5 months and displayed stable disease. Upon disease progression, the patient was treated with aumolertinib as second‐line treatment due to the detection of *EGFR* L858R and T790M mutations, but the symptoms of brain metastases deteriorated. Switching to osimertinib successfully relieved the patient's symptoms and achieved a long progression‐free survival (PFS) of nearly 2 years. Leptomeningeal metastasis was then definitively diagnosed, and the patient eventually died approximately 4 months after osimertinib resistance. This case suggests that osimertinib may be a viable option for *EGFR*‐mutant NSCLC patients after aumolertinib failure, especially for those with intracranial metastases.

**Summary**

- One advanced NSCLC patient with multiple brain metastases was treated with aumolertinib because of *EGFR* L858R and T790M mutations.
- However, the symptoms of cerebral metastases deteriorated.
- Switching to osimertinib successfully relieved the patient's symptoms and achieved a long progression‐free survival (PFS) of nearly 2 years.

## 1 Introduction

Lung cancer, the leading cause of cancer‐related death worldwide, is classified into small cell lung cancer (SCLC) and non‐small cell lung cancer (NSCLC) according to cell morphology [1]. NSCLC is the most common type, accounting for 85% of cases [2]. Epidermal growth factor receptor (EGFR) is one of the most critical driver genes for NSCLC, and *EGFR* mutations are detected in 40%–60% of East Asian patients [3]. Molecular targeted therapies using EGFR tyrosine kinase inhibitors (TKIs) have dramatically improved the prognosis and have become the standard treatment for NSCLC patients with *EGFR* mutations. To date, three generations of EGFR‐TKIs have been developed and approved to target sensitizing *EGFR* mutations, usually referring to exon 19 deletions and exon 21 L858R point mutations. However, only third‐generation EGFR‐TKIs provide benefits for patients with T790M mutations by irreversibly binding to the cysteine‐797 residue in the ATP binding site of EGFR kinase [4].

Osimertinib, a third‐generation TKI, is distinguished by its optimized quinazoline core with a pyrimidine substituent. It is approved by the US Food and Drug Administration (FDA) and the National Medical Products Administration (NMPA) in China for the treatment of *EGFR* T790M mutation‐positive NSCLC, based on data from the AURA3 trial [5]. Aumolertinib, another third‐generation EGFR‐TKI, is derived from the skeleton structure of osimertinib, with a cyclopropyl group substituting the methyl group on the indole ring [6]. This structural element enhances its affinity and selectivity for mutated EGFR kinases, thereby overcoming T790M‐mediated resistance. The Chinese Society of Clinical Oncology (CSCO) guidelines recommend aumolertinib as an alternative treatment for patients with *EGFR* T790M‐positive NSCLC, based on results from the APOLLO trial [7].

Both osimertinib and aumolertinib show higher lipophilicity and enhanced capacity to penetrate the blood–brain barrier (BBB) compared to earlier generations of EGFR‐TKIs, which translates into significant clinical benefits for patients with central nervous system (CNS) metastases, as demonstrated in multiple clinical trials [8, 9, 10]. However, it remains unclear whether structural differences between these two EGFR‐TKIs lead to differences in efficacy, as head‐to‐head comparison trials are unlikely to be conducted [11, 12, 13]. Therefore, real‐world data may provide important insights into selecting the most appropriate EGFR‐TKI.

Here, we present a case of an advanced NSCLC patient with *EGFR* L858R and T790M mutations who responded favorably to osimertinib after resistance to chemotherapy and aumolertinib. This report suggests that osimertinib may be considered as an option for patients with meningeal metastases resistant to aumolertinib.

## 2 Methods

### 2.1 Patient Information

The patient was treated and evaluated at Shangyou County People's Hospital. Tissue and liquid samples were collected for analysis. The patient's son provided written informed consent and permission to use the patient's samples.

### 2.2 Pathology Evaluation

Tissue sediments were fixed in 4% formaldehyde and embedded in paraffin. The 3 μm sections were stained with hematoxylin and eosin (H&E) and immunohistochemistry (IHC). IHC staining was performed using a Real Envision Kit (K5007; Dako, Carpinteria, CA, USA) on an automated immunostaining module (Dako). Antibodies and staining results are detailed in Table 1. Appropriate positive and negative controls were included for each antibody. Staining intensity was classified as follows: −, all tumor cells were negative; −/+, few tumor cells were positive; +, ≥ 5% of tumor cells were positive. Tumor cells showed positive membrane/cytoplasmic staining for CK, positive cytoplasmic staining for Napsin A, negative cytoplasmic staining for Syn and CgA, positive nuclear staining for TTF‐1, and negative nuclear staining for P63 and P40. Cerebrospinal fluid was collected and centrifuged to obtain sufficient cells for cytological analysis. May‐Grünwald–Giemsa staining was widely used for the detection of malignant cells [14] (Table 1).

**TABLE 1:** Staining techniques and results.

Staining techniques	Antibody	Results
Hematoxylin and Eosin (HE) staining	None	Cord‐like/glandular‐like arrangement of atypical cells with large and hyperchromatic nuclei and irregular nuclear shapes
Immunohistochemical staining (IHC)	CK	−
	Napsin A	+
	Syn	−
	CgA	−
	TTF‐1	+
	P63	−
	P40	−
May‐Grünwald–Giemsa (MGG) staining	None	Atypical cells with enlarged nuclei, irregular karyotypes and a high nuclear to cytoplasmic ratio, which are suspected to be adenocarcinoma cells

### 2.3 Next‐Generation Sequencing (NGS), droplet Digital Polymerase Chain Reaction (PCR), and MALDI‐TOF MS

Genomic DNA from formalin‐fixed, paraffin‐embedded (FFPE) tumor tissues or whole blood control samples was extracted using the QIAamp DNA FFPE Tissue Kit (Qiagen) and the DNeasy Blood and tissue Kit (Qiagen), respectively. The DNA was fragmented into approximately 250 bp by the M220 focused‐ultrasonicator (Covaris). Next‐generation sequencing was performed by ThorGene (Beijing) Technology Co. Ltd. (https://www.thorgene.cn). For droplet digital PCR to detect *EGFR* mutations, the experiments were conducted at Amoy Diagnostics Co. Ltd. (Xiamen, China), and the method was reported previously [15]. MALDI‐TOF MS was used to detect *EGFR* mutations according to the user manual of the MassARRAY system (Sequenom, San Diego, CA) and was conducted at Genowise Biotechnology Co. Ltd. (Suzhou, China).

## 3 Case Presentation

**FIGURE 1:**
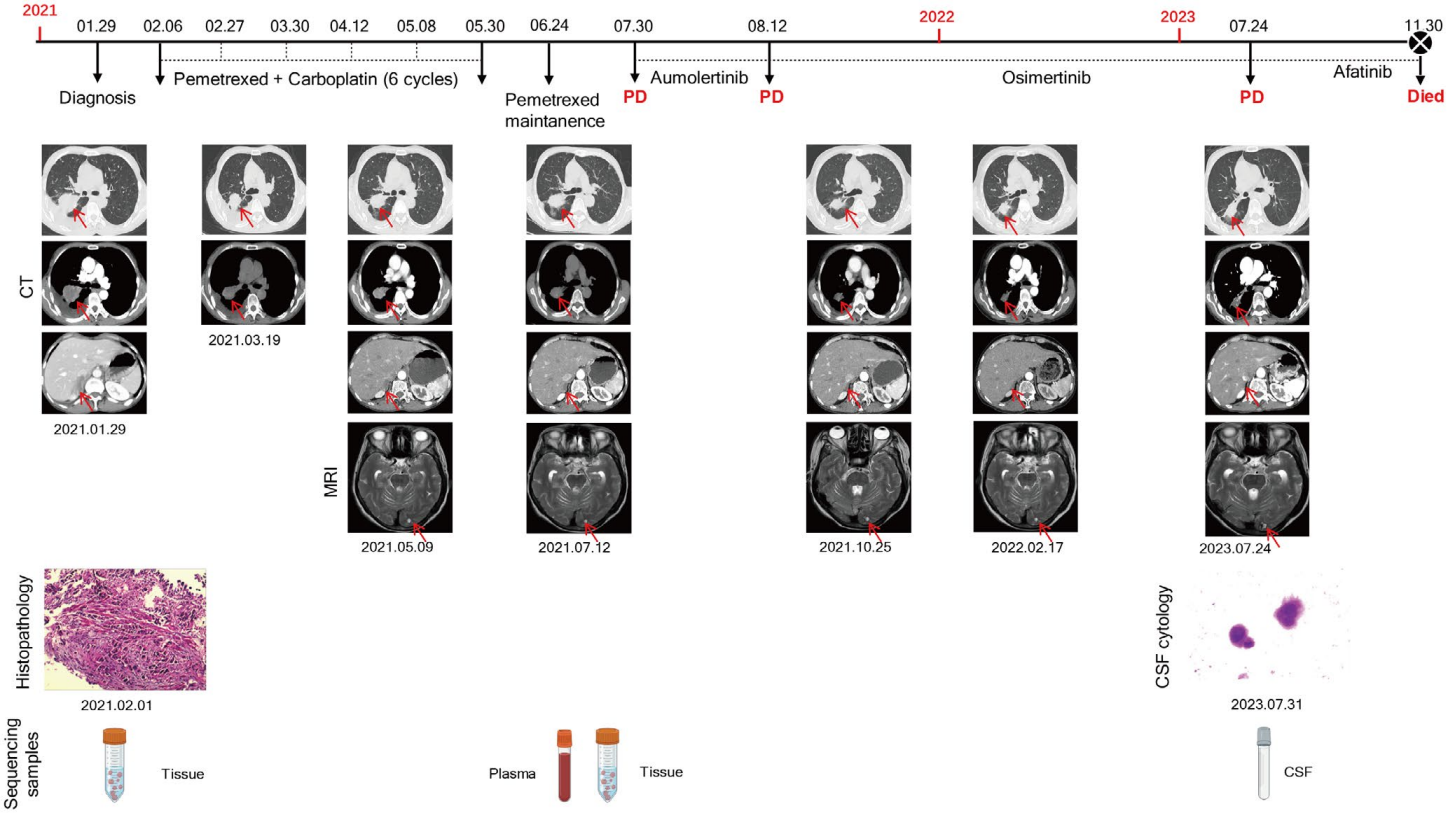
Clinical history of the case, including treatment details, radiographic and pathological findings, and plasma and CSF acquistions. CSF, cerebrospinal fluid; CT, computed tomography; MRI, magnetic resonance imaging; PD, progressed disease.

A 59‐year‐old female patient consulted the People's Hospital of Shangyou County with complaints of a cough lasting more than 2 months and a headache for over 1 week. Her medical history included Grade 2 hypertension for 10 years, with no history of smoking. Her performance status (PS) was assessed as 1 using the Eastern Cooperative Oncology Group (ECOG) score. A computed tomography (CT) scan of the chest and abdomen showed a 48 mm × 49 mm × 52 mm mass occupying the hilus of the right lung, diffuse nodules in both lungs, pleural effusion, right adrenal gland nodules, mediastinal enlarged lymph nodes, and multiple bone destructions. The brain magnetic resonance imaging (MRI) examination revealed multiple metastases in the bilateral occipital lobe. Further bronchoscopy biopsy confirmed the pathological diagnosis of lung adenocarcinoma. According to the American Joint Committee on Cancer (AJCC) version 8.0 for the TNM staging system, the patient was diagnosed with right lung adenocarcinoma at the cT2bN3M1c IVB clinical stage, with lung, adrenal gland, bone, and brain metastases (Figure 1). Considering that the patient was a non‐smoker Asian woman with a relatively high risk of driver gene positivity, NGS with a panel of 10 common genes was recommended on tissue samples from the bronchoscopy biopsy. However, the results were negative for all 10 driver genes (Table 2).

**TABLE 2:** Dynamic genetic sequencing assays and results.

Time	Assay	Sample	Panel	Result threshold
2021‐2‐3	NGS	Tissue	10 genes	Negative 1.0%
2021‐7‐23	Droplet digital PCR	Plasma	EGFR	T790M 0.1%
2021‐7‐25	MALDI‐TOF MS	Tissue	EGFR	L858R 1.0%
2023‐8‐7	NGS	CSF	82 genes	EGFR L858R, 1.0% EGFR L718V

The patient received first‐line chemotherapy with the PC regimen (pemetrexed 500 mg/m^2^ plus carboplatin at an area under the curve of five, administered in a 3‐week cycle for up to 6 cycles, followed by pemetrexed maintenance) and had clinical benefit. Efficacy evaluation showed that the best response was stable disease, with a 14.5% shrinkage from baseline. However, 5 months later, the patient suffered from headache and one‐sided weakness, and her PS worsened to a score of 3. CT and MRI scans showed no evidence of disease progression. Leptomeningeal metastasis was suspected, and a lumbar puncture was performed, but no cancer cells were found in the cerebrospinal fluid (CSF). Considering that the patient is female and a non‐smoker, we suspected false negativity in the initial NGS result and recommended circulating tumor DNA (ctDNA) detection using the blood sample. Because of financial hardship, the patient and her family members chose droplet digital PCR for *EGFR* mutations detection. Unexpectedly, an *EGFR* T790M mutation, the most common resistance mutation to first‐ and second‐generation EGFR‐TKIs, was found in the plasma. At that time, a company promoting MALDI‐TOF MS offered a free test for *EGFR* mutations in tissue samples. To further confirm the gene mutation in the plasma, MALDI‐TOF MS was applied to detect *EGFR* mutations in the initial tissue sample from the bronchoscopy biopsy. The results identified an *EGFR* L858R mutation, a sensitive mutation, with an abundance of 9.79%, while no *EGFR* T790M mutation was detected.

The patient was then treated with aumolertinib 220 mg once daily as second‐line treatment. However, 2 weeks later, the symptoms deteriorated significantly, and new symptoms appeared, including aphagia and aphasia. Leptomeningeal metastasis was still suspected. Considering the meaningful therapeutic efficacy of osimertinib demonstrated in the BLOOM study, we recommended switching the patient to osimertinib. She began osimertinib 160 mg once daily through a nasogastric tube. Only 5 days later, her headache and weakness obviously improved, and she could communicate with her family members. The PS of the patient returned to score 1 after 1 month, and the feeding method transitioned from nasogastric tube feeding to full oral intake. Regular follow‐ups showed gradual recovery in all her symptoms. However, 21 months later, the patient was admitted to the hospital with a recurrence of aphasia and weakness for 1 week. Her PS was assessed as score 3 again. CT and MRI scans showed no evidence of disease progression. Lumbar puncture was performed again, and cancer cells were identified in the CSF. Considering the complex resistance mechanisms of third‐generation EGFR‐TKIs, NGS with a panel of 86 genes was performed. The results detected *EGFR* L858R and L718V mutations but no T790M mutation in CSF cell‐free DNA. Leptomeningeal metastasis was definitely diagnosed. The multidisciplinary team (MDT) discussed this case in detail and recommended the second‐generation EGFR‐TKI afatinib, as previous reports have shown that *EGFR* L718V with L858R is resistant to osimertinib but sensitive to afatinib [16, 17, 18]. The patient was commenced on afatinib 30 mg daily as fourth‐line therapy, and her symptoms stabilized for only 1 month but soon worsened. The family members refused further treatment. The patient suffered from serious headaches and dysphagia and eventually died nearly 4 months after osimertinib resistance.

## 4 Discussion

After primary resistance to aumolertinib, we innovatively switched to osimertinib, successfully relieving the patient's symptoms and achieving a long progression‐free survival (PFS) of nearly 2 years. To our knowledge, this is the first reported case of osimertinib as salvage therapy in a NSCLC patient after aumolertinib resistance, providing a potential new strategy for treating patients with the *EGFR* T790M mutation.

Previous studies report the use of aumolertinib after osimertinib resistance or related toxicity. Shen et al. reported that aumolertinib overcame osimertinib resistance associated with the *EGFR* L718Q mutation in a patient with metastatic NSCLC [19]. In another case report, three patients were treated with aumolertinib after developing osimertinib resistance with the T790M mutation and without other additional genetic mutations. All three achieved a PFS of over 9 months, indicating that aumolertinib may be a viable option after osimertinib failure [20]. Inconsistent with previous publications, the current case had primary resistance to aumolertinib, and the challenge with osimertinib led to significant clinical efficacy and benefits. The underlying mechanisms of this response remain unclear and need further investigation.

An in‐depth understanding of the pharmacological effects of osimertinib and aumolertinib could help analyze the underlying mechanisms. Osimertinib reaches its maximal plasma concentration (*C*_max_) within 6 h after administration, while aumolertinib reaches its *C*_max_ within 4 h. The elimination half‐life (T_1/2_) is 48.6 h for osimertinib and 30.6 h for aumolertinib, respectively [21, 22]. Preclinical BBB models showed that osimertinib achieves significant exposure in the brain of healthy rats, with a total brain‐to‐plasma level ratio (Kp) of 6.1, while the Kp for aumolertinib was only 1.82 in a mouse model, which may partly explain the superior efficacy of osimertinib in this patient [12, 23]. Moreover, in healthy subjects with an intact BBB, osimertinib demonstrated favorable brain penetrance, potentially contributing to its efficacy in treating CNS metastases [24].

In the AURA3 trial, 419 patients with *EGFR* T790M‐positive advanced NSCLC were treated with osimertinib or platinum‐pemetrexed, including 46 patients with measurable CNS lesions. The CNS overall response rate (ORR) of osimertinib was 70%, and the median CNS PFS was 11.7 months [8]. In the phase II APOLLO trial, aumolertinib showed adequate effectiveness in *EGFR* T790M‐positive NSCLC patients, with a CNS ORR of 60.9% and a median CNS PFS of 10.8 months [9]. These results highlight the strong CNS efficacy of both EGFR‐TKIs. Notably, in this case, double doses of aumolertinib and osimertinib were used sequentially. A phase II study evaluated the efficacy of osimertinib 160 mg in T790M‐positive NSCLC patients with brain or leptomeningeal metastases who had progressed on prior EGFR‐TKIs. The intracranial ORR was 55%, with a median PFS of 7.6 months in the brain metastasis cohort and 8.0 months in the leptomeningeal metastasis cohort. Other studies have also proved the meaningful therapeutic efficacy of osimertinib 160 mg, especially in brain and leptomeningeal metastases [10, 25, 26], whereas there was much less data on high‐dose aumolertinib for *EGFR*‐mutant NSCLC patients. Li et al. reported extended effectiveness with increased‐dose aumolertinib in patients whose disease progressed gradually while receiving aumolertinib [27]. The ongoing ACHIEVE study showed a CNS ORR of 86.3% (19/22) in treatment‐naive *EGFR*‐mutant NSCLC patients with asymptomatic brain metastases treated with aumolertinib 165 mg [28]. Another trial comparing high‐dose aumolertinib to osimertinib in *EGFR* T790M‐positive NSCLC patients with brain metastases is ongoing, and its results may provide valuable insights [29]. Currently, the evidence supporting osimertinib 160 mg treatment appears much more robust than that for aumolertinib 220 mg.

Besides, leptomeningeal metastasis was suspected prior to initiating aumolertinib. It is well known that osimertinib demonstrates a promising ORR and survival benefit in *EGFR* T790M‐positive NSCLC patients with leptomeningeal metastases [10, 26, 30]. However, the efficacy of aumolertinib in patients with leptomeningeal involvement remains unclear due to the paucity of clinical data. To date, there is only one case report describing the use of aumolertinib as a single agent for treating *EGFR*‐mutated NSCLC patients with leptomeningeal metastasis, and two studies investigating high‐dose aumolertinib combined with intrathecal chemotherapy, with or without bevacizumab, in patients with leptomeningeal metastases [31, 32, 33]. The limited evidence supporting aumolertinib for the treatment of leptomeningeal metastases may partly explain the superior efficacy of osimertinib observed after aumolertinib failure in this case.

Three different detection strategies of *EGFR* mutations were performed in this case, including NGS, droplet digital PCR, and MALDI‐TOF MS. Previous studies have compared the diagnostic performance, sensitivity, and specificity of these methods. MALDI‐TOF MS allowed the detection of T790M in cases exhibiting mutant‐allele fractions ranging from 11.5% to 17%, while droplet digital PCR and NGS were more sensitive, identifying lower mutant‐allele fractions ranging from 0.07% to 0.38% [34, 35]. The positive rates of *EGFR* T790M detected in plasma samples were 34.2% for droplet digital PCR and 22.5% for NGS [36]. Another study reported a sensitivity of 100% for NGS and 94% for droplet digital PCR, using tissue biopsies as the reference standard [37]. In this case, the initial NGS identified the tumor as *EGFR*‐wild type, which may represent a false negative. However, the potential contribution of spatial tumor heterogeneity cannot be excluded.

There are some caveats to our study. First, the use of different genetic testing modalities at different time points, along with variations in gene panels and detection limits, could introduce biases. Second, as this is a case‐based study, the results should be carefully interpreted. Larger studies with greater sample sizes are warranted to establish clinical efficacy.

## 5 Conclusion

In summary, the current case indicates the potential use of osimertinib as a salvage option for *EGFR*‐mutant NSCLC patients after aumolertinib failure, especially in those with intracranial metastases. Ongoing clinical trials are investigating treatment strategies following resistance to third‐generation EGFR‐TKIs, and this case introduces the possibility of switching to another third‐generation EGFR‐TKI after the failure of one. Most importantly, the exact mechanisms underlying this therapeutic switch need further investigation.

## Author Contributions

**Yongtong Tang:** data curation, investigation, writing – original draft. **Jilan Xu:** data curation, investigation, visualization. **Qingle Chen:** methodology. **Jianping Lu:** validation, visualization. **Li Ren:** methodology. **Taihe Lan:** data curation. **Taihua Liao:** visualization. **Xiaoling Chen:** formal analysis. **Hui Yi:** formal analysis. **Jun Zhang:** conceptualization, methodology. **Jie Huang:** conceptualization, funding acquisition.

## Ethics Statement

This study was approved by the research ethics committee of Guangdong Provincial People's Hospital, Guangdong Academy of Medical Sciences (Ethics number: KY2023‐407‐05).

## Consent

Informed consent was obtained from all individuals included in this study.

## Conflicts of Interest

The authors declare no conflicts of interest.

## Data Availability

Sequencing data can be obtained by contacting the corresponding author upon reasonable request.
